# A brief measure of attitudes toward mixed methods research in psychology

**DOI:** 10.3389/fpsyg.2014.01312

**Published:** 2014-11-12

**Authors:** Lynne D. Roberts, Kate Povee

**Affiliations:** School of Psychology and Speech Pathology, Curtin UniversityPerth, WA, Australia

**Keywords:** attitudes, measure development, mixed methods research, psychology, teaching and learning

## Abstract

The adoption of mixed methods research in psychology has trailed behind other social science disciplines. Teaching psychology students, academics, and practitioners about mixed methodologies may increase the use of mixed methods within the discipline. However, tailoring and evaluating education and training in mixed methodologies requires an understanding of, and way of measuring, attitudes toward mixed methods research in psychology. To date, no such measure exists. In this article we present the development and initial validation of a new measure: *Attitudes toward Mixed Methods Research in Psychology*. A pool of 42 items developed from previous qualitative research on attitudes toward mixed methods research along with validation measures was administered via an online survey to a convenience sample of 274 psychology students, academics and psychologists. Principal axis factoring with varimax rotation on a subset of the sample produced a four-factor, 12-item solution. Confirmatory factor analysis on a separate subset of the sample indicated that a higher order four factor model provided the best fit to the data. The four factors; ‘Limited Exposure,’ ‘(in)Compatibility,’ ‘Validity,’ and ‘Tokenistic Qualitative Component’; each have acceptable internal reliability. Known groups validity analyses based on preferred research orientation and self-rated mixed methods research skills, and convergent and divergent validity analyses based on measures of attitudes toward psychology as a science and scientist and practitioner orientation, provide initial validation of the measure. This brief, internally reliable measure can be used in assessing attitudes toward mixed methods research in psychology, measuring change in attitudes as part of the evaluation of mixed methods education, and in larger research programs.

## INTRODUCTION

The emergence of mixed methods research; the integration of quantitative and qualitative research methods within one project (Johnson et al., 2007); has been heralded as a ‘new era’ (Tashakkori and Creswell, 2007) and ‘the third methodological movement’ (Lopez-Fernandez and Molina-Azorin, 2011) whose ‘time has come’ (Johnson and Onwuegbuzie, 2004). However, the adoption of mixed methods research in psychology has trailed behind other social science disciplines. We begin this article with a brief overview of the prevalence of mixed methods research in the social sciences, before narrowing down to focus on mixed methods research in psychology. We argue that teaching mixed methods research in psychology is central to increasing the use of mixed methods within this field, but that teaching alone is not enough. We need to be able to understand and measure attitudes toward mixed methods research in order to tailor and evaluate education and training in this research methodology. There are currently no measures available to identify and measure changes in attitudes toward mixed methods research in psychology student, academic, and practitioner populations. We present the development and initial validation of a measure of attitudes toward mixed methods research in psychology, building on our earlier research that identified the range of attitudes toward mixed methods research held by psychology students and academics (Povee and Roberts, 2014b). We conclude this article with an evaluation of the psychometric properties of the measure and provide recommendations for its use in assessing attitudes, evaluating the teaching of mixed methods research and in larger research programs.

### MIXED METHODS RESEARCH IN THE SOCIAL SCIENCES

The use of mixed methods research in social sciences is increasing in some disciplines, particularly in applied research areas (Teddlie and Johnson, 2009; Alise and Teddlie, 2010), where mixed methods research may be viewed as a “practical necessity” (Fielding, 2010, p. 127). The proportion of publications featuring mixed methods research in applied social science disciplines (estimated at 16%) is two to three times higher than in ‘pure’ social sciences (including psychology) with an estimated prevalence rate of 6% (Alise and Teddlie, 2010). However, the proportion of published articles reporting mixed methods research varies by journal, even within the same discipline. For example, within education journals, prevalence estimates have ranged widely. Truscott et al. (2010) reviewed articles published in education journals from 1995 to 2005, reporting 14% of articles presented mixed methods research, with no systematic increase across the period. In contrast, Ross and Onwuegbuzie (2010) noted that mixed methods research was reported in 24% of articles published in the *American Educational Research Journal* and 33% of articles published in the *Journal for Research in Mathematics Education* and Alise and Teddlie (2010) reported an estimated prevalence rate of 24% across five elite education journals.

The increased publication rates of mixed methods research in the social sciences has been accompanied by increased scholarly attention to mixed methods as a research methodology. This has included publications on how to conduct mixed methods research (e.g., Tashakkori and Teddlie, 2010; Creswell and Plano-Clark, 2011; Creswell, 2015), how to evaluate mixed methods research (e.g., Leech et al., 2010; O’Cathain, 2010; Collins et al., 2012; Heyvaert et al., 2013), and how to write mixed methods articles (e.g., Leech et al., 2011; Mertens, 2011).

The increased use of mixed methods approaches has also been followed by demand for training and education in using mixed methods research. There is an emerging literature on teaching mixed methods research (e.g., Tashakkori and Teddlie, 2003; Early, 2007; Christ, 2009; Baran, 2010; Ivankova, 2010; Onwuegbuzie et al., 2011, 2013; Poth, 2014), highlighting the difficulties faced by ‘first generation’ mixed methods instructors (Tashakkori and Teddlie, 2003; Early, 2007; Onwuegbuzie et al., 2011), who often have not themselves been trained in mixed methods research (Early, 2007). There is an identified need for dialog and development of resources to support the teaching of mixed methods research (Tashakkori and Teddlie, 2003; Early, 2007).

### MIXED METHODS RESEARCH IN PSYCHOLOGY

The uptake of mixed methods research is lower in psychology journals than many other social science disciplines. Based on systematic random sampling, Alise and Teddlie (2010) estimated that 7% of articles published in five elite psychology journals immediately prior to 2006 presented mixed methods research, with all adopting a quasi-mixed design. Other studies have reported lower prevalence estimates, ranging from 1.7 to 3% (Ponterotto, 2005; Leech and Onwuegbuzie, 2011; Lopez-Fernandez and Molina-Azorin, 2011).

The relative paucity of mixed methods research in psychology is perhaps unsurprising given the historical dominance of behaviorism, positivist, and post-positivist research paradigms and associated valuing of quantitative and experimental methods (Ponterotto, 2005; Alise and Teddlie, 2010), as evidenced by publications in psychology journals, funding, research training, and current teaching models (Walsh-Bowers, 2002; Bhati et al., 2013). Psychology students continue to be socialized within the dominant culture of positivism and quantitative research methods (Breen and Darlaston-Jones, 2010; Bhati et al., 2013). Compounding this, the ‘paradigm wars’ (Gage, 1989) has juxtaposed quantitative and qualitative methods as binary opposites arising from incompatible world views and therefore not suitable for mixing (Wiggins, 2011). Other barriers to conducting mixed methods research include the difficulties in learning and applying both methods (Hanson et al., 2005); particularly given the limited availability of education and training in mixed methods research (O’Cathain et al., 2010). These factors may have contributed to perceptions of lack of rigor in the field (Bergman, 2011).

However, over the last decade, there are encouraging signs of increased interest in mixed methods research in psychology. There have been calls to increase the adoption of this approach to research in psychology (e.g., Haverkamp et al., 2005; Weisner and Fiese, 2011; Barnes, 2012), and the teaching of a wide range of methodologies within the undergraduate psychology curriculum (Breen and Darlaston-Jones, 2010). However, it must be noted that these calls have not been universally welcomed (see, for example, Toomela (2011, p. 38) who argued that qualitative and mixed methods in psychology “may be just other paths to a fairy land; to a land where science and fairy-tales are equally acceptable truths”).

As lack of familiarity with, and expertise in conducting, mixed methods research underlie the low levels of mixed methods research published in psychology journals, teaching mixed methods in psychology is central to increasing the use of mixed methods in psychology. In order to effectively teach mixed methods research in psychology, it is important to understand the attitudes toward mixed methods held by psychology students, academics, and psychologists. As part of a larger mixed methods project examining attitudes toward qualitative and mixed methods research in psychology (see Povee and Roberts, 2014a; Roberts and Povee, 2014 for information on the component of the project examining attitudes toward qualitative research), we interviewed 21 psychology students and academics about their attitudes toward mixed methods research (Povee and Roberts, 2014b). Using the multicomponent model (also known as the tripartite or neotripartite model) of attitudes (Eagly and Chaiken, 1993, 2007) as a framework, through thematic analysis of interview transcripts we identified a range of behavioral and cognitive themes underlying attitudes toward mixed methods research.

The behavioral component of attitudes refers to experience and intentions (Eagly and Chaiken, 1993, 2007). Three themes were identified within the behavior domain. First, mixed methods research was seen as providing opportunities for broadening perspectives of research, sharing knowledge, and learning (‘Expanding Research Capabilities’). Second, a lack of training and experience was seen as limiting opportunities for conducting mixed methods research (‘Limited Exposure’). Third, mixed methods research was viewed as requiring more time, resources and effort than other types of research (‘Time and Resource Intensive’), all barriers to conducting mixed methods research (Povee and Roberts, 2014b).

The cognitive component of attitudes refers to the associations and attributes ascribed to mixed methods research (Eagly and Chaiken, 1993, 2007). Seven themes were identified within the cognitive domain. Mixed methods research was viewed as a flexible approach to psychological inquiry (‘Flexibility’), with the combination of qualitative and quantitative components viewed as either complementary or incompatible [‘(in)Compatibility’]. Mixed Methods research was described by some as being the most ‘valid’ approach to psychological inquiry while others were concerned over the possible mismatch of the findings of the qualitative and quantitative components (‘Validity’). Concern was also raised that the qualitative component of mixed methods research was too often secondary to the quantitative components (‘Tokenistic Qualitative Component’), that mixed methods research may be adopted simply to satisfy quantitatively oriented academics, researchers, or thesis markers (‘Skepticism of Motivation’), that methodologies may be mixed without a considered rationale (‘Rationale for Mixing’) and that a mixed methods approach was more susceptible to researcher bias than purely qualitative or purely quantitative research studies (‘Researcher Bias’).

While understanding the range of attitudes toward mixed methods research held by psychologists and psychology students is helpful in tailoring education and training in mixed methods research, it is also important to be able to evaluate the efficacy of training and evaluation in terms of increased knowledge and skills, and also in terms of attitudes. Previous research on the teaching of research methodologies has demonstrated that increased knowledge is not always associated with increased positive attitudes. For example, research on psychology students’ attitude change with the teaching of quantitative research methods and statistics suggests that while teaching may increase knowledge, attitudes toward the perceived utility of both research methods and statistics decline following teaching (Manning et al., 2006; Sizemore and Lewandowski, 2009). Similar results have been reported across disciplines (Schau and Emmioglu, 2012). Attitudes impact judgments and behaviors (Petty et al., 1997) and if the teaching of mixed methods results in more negative attitudes toward mixed methods research, mixed methods education is unlikely to result in the increased conduct of mixed methods research.

There are currently no measures available to identify and measure changes in attitudes toward mixed methods research in psychology student, academic, and practitioner populations. In this article we draw on the themes identified in our previous research to develop and begin the validation of a new measure to fill this gap: *Attitudes toward Mixed Methods Research in Psychology (AMMRP).* Given the increasing interest in mixed methods research in psychology, such a measure is required to quickly identify attitudes and measure changes in attitudes over time. It may be of particular use within psychology student populations to identify pre-existing attitudes prior to entrance to a course and to measure changes in attitudes after completion of mixed methods research education.

The primary aim of this study was to develop a psychometrically sound brief measure of attitudes toward mixed methods research in psychology. Mixed methods is recommended as the methodology for measure development, particularly Likert scales (Onwuegbuzie et al., 2010), and in our research we used a sequential mixed methods design. Thematic analysis of qualitative interviews (results reported in Povee and Roberts, 2014b) resulted in a pool of items which was then administered to a large sample of psychology students, academics and practitioners. A combination of exploratory and confirmatory factor analyses were used to assess the factor structure of the measure and Cronbach’s alpha calculated to test the internal reliability of factors. This study also begins the validation of the new measure. Known groups validity was examined through comparing scores on the *AMMRP* scales of those who have a stated preference for mixed methods research with those who have a stated preference for qualitative or quantitative research. Convergent and divergent validity was examined through correlations with scores on the ‘Scientist’ and ‘Practitioner’ scales of the *Scientist–Practitioner Inventory for Psychology* (Leong and Zachar, 1991) and the *Psychology as Science Scale* (Friedrich, 1996).

## MATERIALS AND METHODS

A cross-sectional correlational design was utilized with data collected using an online survey.

### PARTICIPANTS

Participants were a convenience sample of 274 psychology students, academics, and practitioners. Reflecting the gender bias in the psychology student and practitioner population, 74.1% of the research participants were female. Participants ranged in age from 18 to 87 (*M* = 28 years SD = 12 years) and resided in a range of countries including Australia (58.8%), the UK (15.6%), the USA (11.5%), and Singapore (5%). The majority were psychology students (78.1%), ranging from first year undergraduate to Ph.D. students. Approximately one-fifth (22.2%) of the sample were academics with between one and 33 years in academia and 11.7% were currently employed as psychologists across a range of specialities^1^. Almost half of participants (46.2%) expressed a preference for conducting mixed methods research, while approximately a quarter each expressed a preference for qualitative (28.4%) and quantitative research (25.4%). Self-rated skills in conducting mixed methods research ranged from very poor (5.6%), poor (21.6%), fair (43.7%), good (24.6%) to very good (4.5%).

### MEASURES

An online questionnaire was constructed containing items designed to measure attitudes to qualitative^2^ and mixed methods research, the *Psychology as Science Scale,* The *Scientist–Practitioner Inventory for Psychology* and a range of demographic questions.

*Attitudes toward Mixed Methods Research in Psychology* items were developed based on the themes identified in our prior qualitative analysis (Povee and Roberts, 2014b). A pool of 42 items (short statements) designed to measure the eleven themes of ‘Expanding Research Capabilities,’ ‘Limited Exposure,’ ‘Time and Resource Intensive,’ ‘Flexibility,’ ‘(in)Compatibility,’ ‘Increased Validity,’ ‘(in)Congruency,’ ‘Tokenistic Qualitative Component,’ ‘Skepticism of Motivation,’ ‘Rationale for Mixing,’ and ‘Researcher Bias’ were developed by the authors. This pool of items was sent to a psychology academic with expertise in research methodologies for expert review. Based on the feedback provided, some items were removed and minor changes were made to other items. The final pool of 34 items was used in the survey. Attitudes are expected to vary in valence (from positive to negative) and strength (from weak to strong; Maio and Haddock, 2010). The items in the item pool vary in valence so a response format was needed that could measure strength. In line with this, a response format of strongly disagree to strongly agree was selected for use with all items.

The *Psychology as Science Scale* (Friedrich, 1996) is a 15-item scale designed to measure perceptions of psychology as a science. Three factors underlie the scale, measuring (a) perceptions of psychology as a hard science (example item “It’s just as important for psychology students to do experiments as it is for students in chemistry and biology”), (b) the perceived value of methodological training and psychological research (example item “Courses in psychology place too much emphasis on research and experimentation”, and (c) deterministic views regarding the predictability of human behavior (example item “Carefully controlled research is not likely to be useful in solving psychological problems”). This measure was selected as it has a bias toward quantitative research in psychology. Each item is rated by participants on a seven point scale ranging from (1) *strongly disagree* to (7) *strongly agree.* Seven items are reverse-scored. Scores range from 15 to 85 with higher scores indicating increased perceptions of psychology as a science. The measure is intended to be used as an overall score (Friedrich, 1996), with the total scale having acceptable internal reliability with samples of undergraduate psychology students (α = 0.71 to 0.72; Friedrich, 1996; Holmes and Beins, 2009). In this sample the internal reliability was good (α = 0.86).

The *Scientist–Practitioner Inventory for Psychology* (Leong and Zachar, 1991) is a 42-item inventory that measures scientist and practitioner interests in psychology. Participants are asked to rate each item in terms of their level of interest in conducting the specified activities in their future careers. The five point response scale ranges from (1) *very low interest* to (5) *very high interest*. Whilst exploratory factor analysis suggests a seven factor solution underlies the items, there are two second order factors and the measure is treated as two separate scales: ‘Scientist’ scale and ‘Practitioner’ scale. Each scale consists of 21 items. Example items are “Collecting data on a research project you designed” (‘Scientist’ scale) and “Conducting group psychotherapy sessions” (‘Practitioner’ scale). Possible scale scores range from 21 to 105, with higher scores representing higher interest. The scales have good internal reliability: ‘Scientist’ scale α = 0.91, ‘Practitioner’ scale α = 0.88 to 0.94 (Leong and Zachar, 1991; Holmes and Beins, 2009). In this sample, the internal reliability was also high (‘Scientist’ scale α = 0.94, ‘Practitioner’ scale α = 0.95).

### PROCEDURE

Prior to commencing the research, ethics approval was obtained from Curtin University Human Research Ethics Committee. Recruitment for the research commenced in July 2012, through messages posted on social networking sites (Facebook; Linked In) and advertisements on noticeboards around the university. Interested persons were provided with a link to an online participant information sheet, and upon consenting to participate were redirected to an online questionnaire Participation in the research was voluntary and participants who completed the survey were offered the opportunity to enter into a draw for a US$100 Amazon.com gift voucher. In addition, students in the second year undergraduate psychology participant pool had the option of participating in this study, and those electing to do so were assigned credits toward their research participation requirement.

Recruitment ceased in December 2012, with survey data downloaded into SPSS v. 20 for analysis. Three hundred and twenty four participants had accessed the online survey. In initial screening 50 cases where the survey had not been commenced or mixed methods items had not been completed were deleted, leaving a data set with 274 cases. To meet the suggested criterion for sample size of five items per variable, 170 cases (5 × 34 variables) were randomly selected and used as the dataset for the exploratory factor analysis. The remaining 104 cases were used as the dataset for the confirmatory factor analysis.

## RESULTS

### EXPLORATORY FACTOR ANALYSIS

The exploratory factor analysis dataset was inspected for missing values. In total there were 16 missing data points across the 34 items (0.002%). These were replaced using mean item scores. Prior to conducting the exploratory factor analysis, a parallel analysis was conducted and indicated five factors should be retained. Based on this, Principal Axis Factoring with varimax rotation was conducted with a forced five factor extraction. Items that cross-loaded across factors with a loading greater than 0.3 on a second factor were removed. This resulted in the removal of the fifth factor, as all items cross-loaded above 0.3 on other factors. An iterative process was used to continue to remove items that cross-loaded, loaded weakly or reduced the internal reliability of the factor. The resulting 12 item four factor model, with factor loadings, is presented in **Table 1**. The items in the first factor reflect the behavioral component of attitudes and relate to the individual’s perceived ability to conduct mixed methods research, reflecting the ‘Limited Exposure’ theme from the qualitative analysis. The second, third, and fourth factors contains items reflecting the ‘(in)Compatibility,’ ‘Validity,’ and ‘Tokenistic Qualitative Component’ themes from the qualitative research (Povee and Roberts, 2014b) and have been named accordingly.

**Table 1:** Factor loadings for exploratory factor analysis with varimax rotation for attitudes to mixed methods.

	Factor			
Item	1	2	3	4
I don’t have the skills to conduct MMR	0.883			
I do not have the knowledge to conduct MMR	0.795			
I do not have the experience to conduct MMR	0.771			
I have the confidence to conduct MMR (R)	-0.714			
Qualitative and quantitative methodologies are incompatible		0.769		
MMR can provide converging evidence (R)		-0.633		
MMR is only useful for developing questionnaires and measures		0.629		
Qualitative and quantitative methodologies should not be combined in the one study		0.616		
You can have more confidence in the findings of MMR than research that just uses qualitative or quantitative data alone			0.897	
MMR is more valid than research that just uses qualitative or quantitative data alone			0.839	
In MMR, a small qualitative component is often just ‘tacked on’ to a quantitative study				0.757
The use of qualitative methods in a mixed methods design is tokenistic				0.687

*MMR, Mixed Methods Research; (R), recoded; Factor loadings less than 0.3 suppressed, Factor 1, Limited Exposure; Factor 2, (in)compatability; Factor 3, ‘Increased Validity’; Factor 4, Tokenistic Qualitative Component.*

### CONFIRMATORY FACTOR ANALYSIS

**FIGURE 1:**
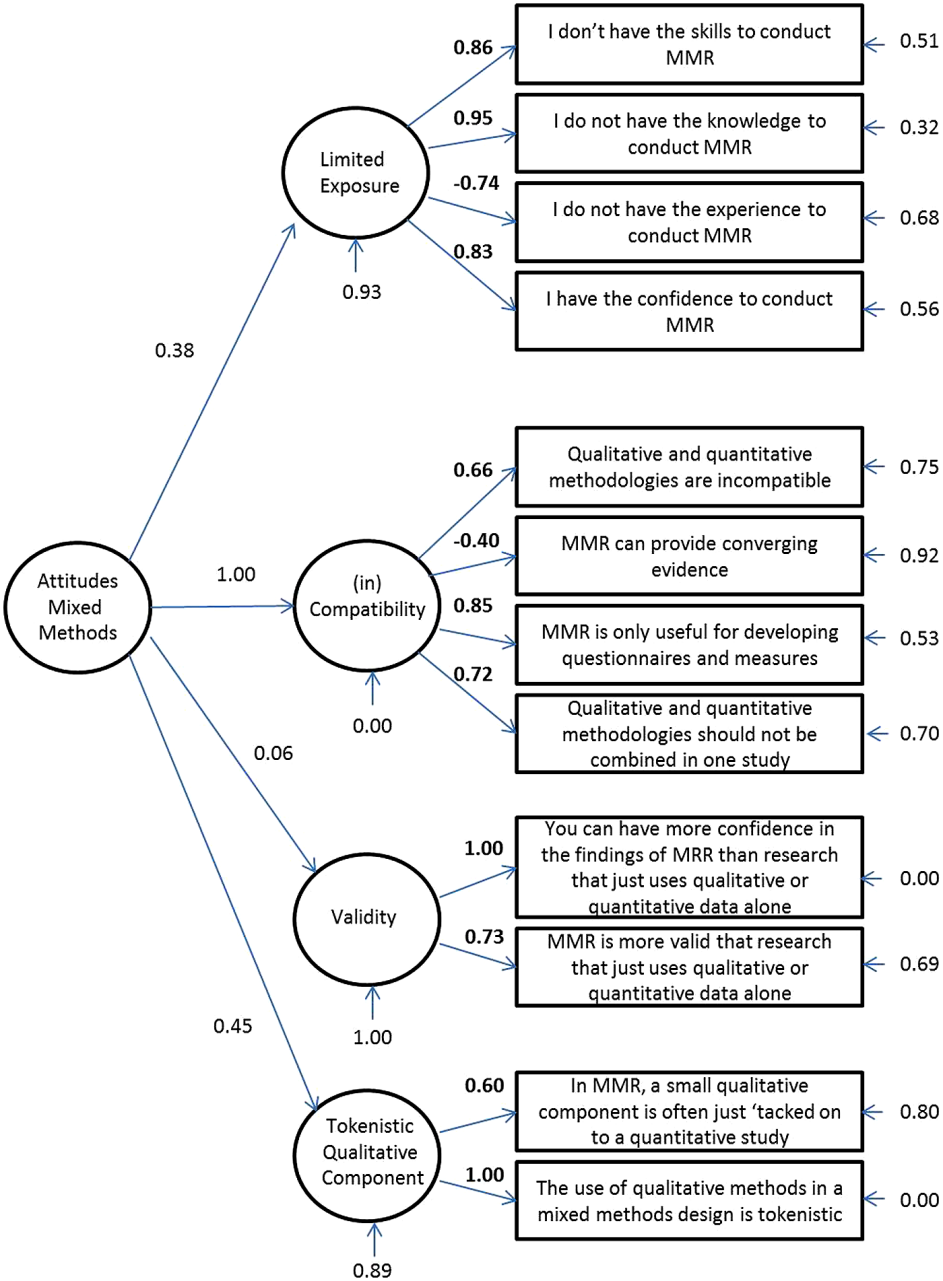
**Confirmatory factor analyses**.

The remaining 104 cases were used as the sample for conducting confirmatory factor analysis using EQS v. 6.2. This dataset was inspected for missing values and item means were used to substitute the two missing data points. A higher-order four factor model and uncorrelated four factor model were tested against a single factor model for goodness of fit using the recommended cut-offs for four fit indices: the Satorra-Bentler Chi Square divided by degrees of freedom, the comparative fit index (CFI), the non-normed fit index (NNFI), and the root mean square error of approximation (RMSEA). The fit indices for each model are presented in **Table 2**, and clearly indicate that the four factor higher order model is preferred as it is the only model that meets all the recommended cut-off criteria for good fit. The four factor higher order model is presented in **Figure 1**.

**Table 2:** Fit indices (robust statistics) for confirmatory factor analysis models of the attitudes to mixed methods measure.

Model cut-off criteria	S-B χ^2^/df *p* > 0.05	CFI≥0.85	NNFI ≥0.85	RMSEA ≤0.06
One Factor Model	0.000	0.566	0.470	0.195
Uncorrelated 4 Factor Model	0.003	0.933	0.918	0.077
Higher Order 4 Factor Model	0.103	0.974	0.964	0.050

*S-B, Satorra-Bentler; CFI, comparative fit index; NNFI, non-normed fit index; RMSEA, Root Mean Square Error of Approximation.*

### INTERNAL RELIABILITY AND VALIDITY ANALYSES

The two datasets were merged for internal reliability and validity analyses. The internal reliability coefficients for each factor in the exploratory factor analysis data set (data set 1), confirmatory factor analysis data set (data set 2), and merged data set (data set 3) are presented in **Table 3**. All factors have acceptable internal reliability. Descriptive statistics for the new measure are presented in **Table 4** and the correlation between factors in **Table 5**.

**Table 3:** Internal reliability of factors (Cronbach’s alpha coefficients).

Factor	Data set 1 α	Data set 2 α	Data set 3 α
Limited exposure	0.88	0.91	0.89
(in)Compatibility	0.77	0.75	0.76
Validity	0.87	0.83	0.86
Tokenistic Qualitative Component	0.69	0.74	0.71

**Table 4:** Descriptive statistics for the attitudes toward mixed methods research in psychology measure.

Scale	Mean (SD)	Possible range	Actual range
Limited exposure	3.03(.89)	1.00–5.00	1.00–5.00
(in)Compatibility	2.09(.65)	1.00–5.00	1.00–4.50
Validity	3.22(.93)	1.00–5.00	1.00–5.00
Tokenistic Qualitative Component	2.80(.73)	1.00–5.00	1.00–5.00

*^a^Higher scores represent lower levels of exposure.*

*^b^Higher scores represent greater perceptions of incompatibility.*

*^c^Higher scores represent greater perceptions of validity.*

*^d^Higher scores represent greater perceptions of a tokenistic qualitative oxcomponent.*

**Table 5:** Correlations of factors.

Scale	Limited exposure	(in)Compatibility	Validity	Tokenistic
Limited exposure	1.00			
(in)Compatibility	0.287*	1.00		
Validity	0.083	-0.094	1.00	
Tokenistic Qualitative Component	-0.007	0.282*	-0.002	1.00

*p < 0.01.

A conservative alpha of *p* < 0.01 was adopted for all validity tests. Known groups validity was examined through comparing scores on the *AMMRP* scales of those who have a stated preference for mixed methods research with those who have a stated preference for qualitative or mixed methods research. Four one way ANOVAs with planned contrasts were conducted and the results are presented in **Table 6**. While those preferring mixed methods research did not significantly differ on ‘Limited Exposure’ to mixed methods or perceptions of a ‘Tokenistic Qualitative Component,’ they were less likely to view the mixing of qualitative and quantitative components as problematic [‘(in)Compatibility’], and more likely to view mixed methods as a valid methodology (‘Validity’).

**Table 6:** AMMRP scale scores by preferred research methodology (*N* = 264).

Preferred methodology	*N*	M (SD)	Lower CI	Upper CI	*F*	Sig	Effect Size (*d*)
*Limited Exposure*					1.48	0.230	0.11
Quantitative	67	3.18(0.91)	2.95	3.40			
Qualitative	75	3.02(0.79)	2.84	3.20			
Mixed Methods	122	2.94(0.95)	2.77	3.11			
*(in)Compatibility*					5.72	0.004*	0.21
Quantitative	67	2.16(0.70)	1.99	2.33			
Qualitative	75	2.24(0.69)	2.08	2.40			
Mixed methods	122	1.94(0.56)	1.84	2.04			
*Validity*					8.71	0.000*	0.26
Quantitative	67	2.96(0.90)	2.74	3.18			
Qualitative	75	3.05(1.01)	2.81	3.28			
Mixed methods	122	3.47(0.85)	3.32	3.62			
*Tokenistic Qualitative Component*					3.10	0.047	0.15
Quantitative	67	2.84(0.85)	2.63	3.04			
Qualitative	75	2.93(0.72)	2.77	3.10			
Mixed methods	122	2.68(0.66)	2.56	2.79			

*Significant planned contrasts *p* < 0.01.

Respondents’ self-ratings of mixed methods skills (measured on a 5 point scale ranging from ‘*very poor*’ to ‘*very good*’) were correlated with *AMMRP* scale scores. The results indicate that there is a strong negative correlation (*r*_S_ = -0.57, *p* < 0.001, *N* = 268) between self-rated mixed methods skills and ‘Limited Exposure,’ and weaker correlations with ‘(in)Compatibility’(*r*_S_ = -0.22, *p* < 0.001, *N* = 268) and ‘Validity’ (*r*_S_ = 0.15, *p* < 0.001, *N* = 268). There was no significant relationship between self-rated skills and the ‘Tokenistic Qualitative Component’ scale.

Independent samples *t*-tests revealed that students (*N* = 211) scored significantly higher than other survey respondents (*N* = 59) on the ‘Limited Exposure’ (students *M* = 3.23, SD = 0.80; non-students *M* = 2.30, SD = 0.88; *t*(268) = 7.725, *p* < 0.001, *d* = 0.81) and ‘(in)Compatibility’ scales (students *M* = 2.15, SD = 0.61; non-students *M* = 1.88, SD = 0.73; *t*(268) = 2.862, *p* = 0.005, *d* = 0.64). However, they did not differ on the ‘Validity’ (students *M* = 3.29, SD = 0.88; non-students *M* = 2.98, SD = 1.08; *t*(268) = 2.315, *p* = 0.021, *d* = 0.93.) or ‘Tokenistic Qualitative Component’ scales (students *M* = 2.75, SD = 0.69; non-students *M* = 2.93, SD = 0.85; *t*(80.56) = -1.50, *p* = 0.136, *d* = 0.73). Students responses on the *AMMRP* scales varied by year of study, with significant negative correlations between year of study and ‘Limited Exposure’ (*r_S_* = -0.431, *p* < 0.001) and ‘(in)Compatibility’ (*r_S_* = -0.406, *p* < 0.001).

Convergent and divergent validity were examined through correlating scores on the newly developed *AMMRP* scales with the ‘Scientist’ and ‘Practitioner’ scales of the *Scientist–Practitioner Inventory for Psychology* (Leong and Zachar, 1991) and the *Psychology as Science Scale* (Friedrich, 1996). The results are presented in **Table 7**. Scores on the ‘(in)Compatibility’ scale were negatively associated with scores on both the ‘Scientist’ scale of the *Scientist–Practitioner Inventory for Psychology* and *Psychology as Science Scale*. The strongest negative relationship (large effect size) was found between ‘(in)Compatibility’ and *Psychology as Science Scale*. Consistent with this, scores on the ‘(in)Compatibility’ scale were also negatively associated with scores on the ‘Scientist’ scale of the *Scientist–Practitioner Inventory for Psychology* (small to medium effect size). ‘Limited exposure’ was positively associated with the ‘Practitioner’ scale, but negatively correlated with the ‘Scientist’ scale of the *Scientist–Practitioner Inventory for Psychology* (small to medium size correlations). ‘Tokenistic Qualitative Component’ was negatively correlated with both the ‘Practitioner’ scale of the *Scientist–Practitioner Inventory for Psychology* and *Psychology as Science Scale* (small to medium size correlations). ‘Validity’ was weakly correlated with *Psychology as Science Scale*, but neither the ‘Scientist’ nor ‘Practitioner’ scales of the *Scientist–Practitioner Inventory for Psychology*.

**Table 7:** Convergent and divergent validity of AMMRP scales.

	Scientist	Practitioner	Psychology as a Science
Limited exposure	-0.327*	0.240*	0.003
(in)Compatibility	-0.200*	0.061	-0.497*
Validity	-0.019	0.104	0.164*
Tokenistic Qualitative Component	0.055	-0.190*	-0.257*

*p < 0.01.

## DISCUSSION

In this paper we have presented the development and initial validation of a new brief measure of attitudes toward mixed methods research in psychology, the *AMMRP*. This measure has good content validity, covering four themes that emerged in previous qualitative research (Povee and Roberts, 2014b). The converging results of the exploratory factor analysis and confirmatory factor analysis support the factor structure of the measure, with confirmatory factor analysis indicating that a higher order four factor model best represented the measure. Internal reliability testing indicated the scales have good internal consistency.

Supporting the validity of the measure, known groups analyses demonstrated that respondents indicating a preference for mixed methods research rated the mixing of qualitative and quantitative components as less ‘(in)Compatible,’ and mixed methods research as higher in ‘Validity’ than respondents with a preference for qualitative or quantitative research. Perhaps surprisingly, a preference for mixed methods research was not significantly associated with ‘Limited Exposure.’ This is however, consistent with previous findings that training in a particular type of research methodology, while increasing skill levels, may have a negative effect on the perceived utility of the research method (Manning et al., 2006; Sizemore and Lewandowski, 2009). Self-ratings of mixed methods skills were strongly negatively related to scores on the ‘Limited Exposure’ scale, in addition to being negatively related to ‘(in)Compatibility’ and positively related to ‘Validity,’ further supporting the known groups validity of the measure.

Students scored lower on the ‘Limited Exposure’ scale than other respondents, with a negative relationship also found between year of study and ‘Limited Exposure’ scores. Similarly, students rated combining qualitative and quantitative methods more problematic [‘(in)Compatibility scale’] than non-students, but the relationship with year of study was negative. Combined, these results suggest that as psychology students’ progress through their undergraduate and postgraduate studies their exposure to mixed methods increases and their perceptions of incompatibility decrease. This is a positive finding for the future of mixed methods research within the discipline.

In the absence of an existing ‘gold standard’ measure of attitudes to mixed methods research, divergent, and convergent validity was assessed through associations with scientist and practitioner measures and a measure of psychology as a science. Divergent validity was demonstrated through the absence of large correlations between *AMMRP* scales and the three other measures. Interestingly, ‘(in)Compatibility’ and ‘Tokenistic Qualitative Component’ were negatively correlated, and ‘Validity’ positively correlated with perceptions of psychology as a science. This indicates that in this sample mixed methods research was viewed as compatible with a belief in psychology as a science.

A limitation of this study is the use of convenience sampling. Given the limited amount of mixed methods research currently published in psychology journals (Ponterotto, 2005; Alise and Teddlie, 2010; Leech and Onwuegbuzie, 2011; Lopez-Fernandez and Molina-Azorin, 2011) a surprisingly large proportion of respondents (almost half of the sample) nominated mixed methods research as their preferred methodology. It may be that the topic of this research attracted those students/academics/practitioners with an interest in mixed methods approaches and as such, the sample cannot be seen as representative of the wider psychology student/academic/practitioner population. Despite this, our survey respondents self-rated mixed methods research skills were normally distributed over the full range of possible scores, and scores on each of the *AMMRP* scales were moderate, covering the full range of possible scores, increasing our confidence in our findings. However, future research conducted with a larger, more representative sample is encouraged to examine the stability of the factor structure of the *AMMRP*. This will also enable further testing of the relationships between AMMRP scales and validity measures as, despite the adoption of a conservative alpha of *p* < 0.01 for validity testing, there remains the possibility of type 1 errors associated with multiple testing.

The promising psychometric properties and brevity of the *AMMRP* indicate its suitability for use in teaching, evaluation, and research. The *AMMRP* administered at the start of a mixed methods course can provide instructors with information on common misperceptions of mixed methods research which can be targeted during teaching. Administered again at the end of the course, the AMMRP can be used as a measure of changing attitudes, evaluating the effectiveness of mixed methods training in changing attitudes toward mixed methods research in psychology. The *AMMRP* is also suitable for use in research projects investigating attitudes toward mixed methods research as a predictor or outcome of other variables of interest. There now exists a full suite of measures assessing attitudes toward research: *AMMRP*, *Attitudes Toward Qualitative Research in Psychology* (*ATQRP*; Roberts and Povee, 2014) and *Psychology as a Science* (Friedrich, 1996). Used together, these three measures will allow a more nuanced assessment of attitudes toward psychological research.

In summary, this paper has presented the development and initial validation of a 12-item measure of attitudes toward mixed methods research in psychology, the *AMMRP.* This is a brief, internally reliable measure that can be used in assessing attitudes toward mixed methods research in psychology and measuring change in attitudes over time.

## Conflict of Interest Statement

The authors declare that the research was conducted in the absence of any commercial or financial relationships that could be construed as a potential conflict of interest.

## Footnotes

1 Some respondents fell into multiple categories (e.g., academic and practicing psychologist).

2 Only the mixed methods items are of interest in this paper. Details of the results for qualitative items have been reported in Roberts and Povee (2014).
